# Validity of Hydration Non-Invasive Indices during the Weightcutting and Official Weigh-In for Olympic Combat Sports

**DOI:** 10.1371/journal.pone.0095336

**Published:** 2014-04-16

**Authors:** Valentín E. Fernández-Elías, Alberto Martínez-Abellán, José María López-Gullón, Ricardo Morán-Navarro, Jesús G. Pallarés, Ernesto De la Cruz-Sánchez, Ricardo Mora-Rodriguez

**Affiliations:** 1 Exercise Physiology Laboratory, University of Castilla-La Mancha, Toledo, Spain; 2 Department of Physical Activity and Sport, University of Murcia, Murcia, Spain; University of Sao Paulo, Brazil

## Abstract

**Background:**

In Olympic combat sports, weight cutting is a common practice aimed to take advantage of competing in weight divisions below the athlete's normal weight. Fluid and food restriction in combination with dehydration (sauna and/or exercise induced profuse sweating) are common weight cut methods. However, the resultant hypohydration could adversely affect health and performance outcomes.

**Purpose:**

The aim of this study is to determine which of the routinely used non-invasive measures of dehydration best track urine osmolality, the gold standard non-invasive test.

**Method:**

Immediately prior to the official weigh-in of three National Championships, the hydration status of 345 athletes of Olympic combat sports (i.e., taekwondo, boxing and wrestling) was determined using five separate techniques: *i*) urine osmolality (U_OSM_), *ii*) urine specific gravity (U_SG_), *iii*) urine color (U_COL_), *iv*) bioelectrical impedance analysis (BIA), and *v*) thirst perception scale (TPS). All techniques were correlated with U_OSM_ divided into three groups: euhydrated (G_1_; U_OSM_ 250–700 mOsm·kg H_2_O^−1^), dehydrated (G_2_; U_OSM_ 701–1080 mOsm·kg H_2_O^−1^), and severely dehydrated (G_3_; U_OSM_ 1081–1500 mOsm·kg H_2_O^−1^).

**Results:**

We found a positive high correlation between the U_OSM_ and U_SG_ (r = 0.89: p = 0.000), although this relationship lost strength as dehydration increased (G_1_ r = 0.92; G_2_ r = 0.73; and G_3_ r = 0.65; p = 0.000). U_COL_ showed a moderate although significant correlation when considering the whole sample (r = 0.743: p = 0.000) and G_1_ (r = 0.702: p = 0.000) but low correlation for the two dehydrated groups (r = 0.498–0.398). TPS and BIA showed very low correlation sizes for all groups assessed.

**Conclusion:**

In a wide range of pre-competitive hydration status (U_OSM_ 250–1500 mOsm·kg H_2_O^−1^), U_SG_ is highly associated with U_OSM_ while being a more affordable and easy to use technique. U_COL_ is a suitable tool when U_SG_ is not available. However, BIA or TPS are not sensitive enough to detect hypohydration at official weight-in before an Olympic combat championship.

## Introduction

Severe dehydration has physiological consequences negatively affecting health and athletic performance. Body water losses exceeding 2% of body weight reduce physical work capacity and exercise performance [1]–[3] and higher dehydration levels (i.e.>4–5%) has been reported to increase heat-stroke risk [1], [4]. These adverse effects include impaired glycogen use [9], increases in core temperature inducing central nervous system fatigue [10], [11], cardiovascular strain [12], [13] and loss of efficacy of the metabolic acid buffer system [14]. All these effects could compromise health and physical performance in military personnel, firemen, athletes training and competing in hot environments, or those involved in Olympic weight-class sports (e.g. wrestling, boxing, judo, taekwondo and weightlifting). In these sports weight loss throughout dehydration is a very common strategy prior to competition [15]. Weight loss by dehydration has been shown to affect boxing and wrestling performance [5], [6]. If that weight loss is quickly recovered the effects on performance are not evident [7], [8]. Many techniques are available to assess body water deficit, however it is not clear which it is best to use in a pre-competition setting. Ideally, this should be a non-invasive index, as well as being fast, accurate, inexpensive and easy-to-use.

Out of the available techniques to measure hydration status, blood osmolality is the gold standard [16]–[18]. However, the measurement of blood osmolality requires an invasive technique, costly measurement apparatus and qualified personnel to handle blood. All these conditions are rarely available to scientists and coaches at the field. Urine analysis of hydration status has been recommended as an alternative measurement because it involves a noninvasive evaluation of body fluid [19]. The main criticism of the use urine as an index of dehydration is that urine does not respond as fast or as accurately as blood to body fluid deficit [16]. However, we have recently found that urine readily tracks blood responses during progressive dehydration induced by exercise [20], [21]. Urine can be analyzed for color, density, osmolality or its constituents resulting in a wide range of hydration indexes. Nonetheless, not all indexes are adequate, accurate or practical, and some are costly and require technical expertise [22].

A non-invasive surrogate of blood osmolality is urine osmolality (U_OSM_) considered the most valid measurement of hydration status through urine [16], [23]. However, similarly to blood osmolality it requires expensive biochemical analysis. Urine specific gravity (U_SG_) assessment requires a simpler apparatus (i.e., refractrometer). Some authors have found that U_SG_ [20], [21], [24]and urine color (U_COL_) [22], [25] are highly correlated to urine osmolality (U_OSM_). Armstrong and co-workers, found acceptable validity of U_SG_ and color analysis in different populations at moderate dehydration levels [17]. However, the agreement between these urine indexes after severe dehydration in weight class sports [15], has not been reported.

Finally, there are non-invasive indexes that do not entail urine collection and analysis. Bioelectrical impedance analysis (BIA; [26]–[29]) and thirst perception scale (TPS; [30]–[33]) have been proposed as simpler indexes of body fluid deficit. Despite all these studies, to our knowledge, there is insufficient evidence to decide about the suitability of these indexes to readily detect whole body dehydration. Furthermore, these indexes have not been evaluated in a large population of athletes undergoing different degrees of dehydration. We believe that a good test for BIA and TPS will be to assess its agreement with U_OSM_ during the weight cutting in Olympic combat sports.

Therefore, the purpose of this study was to compare several non-invasive indexes of hydration in a large number of Olympic combat sport athletes undergoing different degrees of weight loss by dehydration before a real competition. Our intention is to obtain a wide range of hypohydration levels to fully evaluate the detection power of all indexes in comparison to U_OSM_. We hypothesized that techniques involving urine analysis may have high levels of agreement while other estimations (i.e. BIA and TPS) will not.

## Methods

### Participants

Two hundred and forty-four male (age 22.8±4.1 yr, body mass 74.1±15.1 kg, height 176.1±6.7 cm) and one hundred one female (age 22.7±4.5 yr, body mass 57.1±8.9 kg, height 164.9±7.2 cm) high performance athletes of three different Olympic combat sports volunteered to participate in this study: wrestling (n = 157), taekwondo (n = 152) and boxing (n = 36). All participants had at least 4years of training and competition experience, and all of them made the weight in the official weigh-in of their respective national championship during the experimental phase of this study. The subjects and coaches were informed in detail about the experimental procedures and the possible risks and benefits of the project. The study, which complied with the Declaration of Helsinki, was approved by the Bioethics Commission of the University of Murcia, and written informed consent was obtained from athletes prior to participation.

### Study design and experimental protocol

Athletes' hydration status was evaluated through 5 different techniques (i.e., U_OSMO_, U_SG_, U_COL_, TPS and BIA) between 60 and 5 minutes before the official weigh-in of their respective National Championship. No instructions were given to athletes or their coaches about their weight control management. Participants filled out a nutritional questionnaire and twelve of them were excluded from the study for being ingesting vitamins, nutritional supplements or prescription drugs prone to alter urine color, amount or composition [25]. Women were tested out of the proliferative phase of their menstruation.

At arrival to the official weigh-in facilities, a 10 ml mid flow urine sample was obtained from each athlete. After the recipient with the urine sample was handed over and codified, subjects filled out the thirst perception scale, and their body impedance was determined using a Bio-impedance analyzer. Urine specimens were immediately analyzed in duplicate for urine osmolality (U_OSM_), urine specific gravity (U_SG_), and urine color (U_COL_) by the same experienced investigator. The final value for each assessment was the average of the two trials.

#### Urine osmolality

U_OSM_ is the measure of the total urine solute content. As has been repeatedly reported [16], [23], we considered this assessment as our gold standard measurement to determine the athletes' hydration status. Athletes urine specimens (20 µL) were immediately analyzed in duplicate by freezing point depression osmometry (Model 3250, Advanced Instruments, USA).

#### Urine specific gravity

U_SG_ is the analysis of urine density compared to double distilled water (density = 1.000). After apparatus calibration and thorough mixing of the urine specimen, a few drops were placed on the refractometer (URC-NE, Atago, Japan) visor and U_SG_ was determined.

#### Urine color

U_COL_ is determined by the amount of urochrome present in the urine specimen. When large volumes of urine are excreted, the urine is dilute and pale. Conversely, when small volumes of urine are excreted, the urine is concentrated and dark [23]. U_COL_ was determined as described by Arsmstrong et al., [17], [18], [22], [23], [25]. Briefly, an 8 number scale ranging from very pale yellow (number 1) to brownish green (number 8), was used. U_COL_ was determined in duplicate by holding each specimen container next to a validated color scale in a well-lit room.

#### Bioelectrical impedance analysis

BIA has the potential to assess changes in hydration status and has been previously used and validated in combat sports athletes [29]. Athletes BIA was determined using an 8-contact electrode segmental and mono-frequency body composition analyzer (Tanita BC-418, Tanita Corp., Tokyo, Japan) while they were barefoot, wearing shorts and a sports-top for females.

#### Thirst perception scale (TPS)

Thirst perception is physiologically related to the hydration status of an individual since it is mediated by fluid-regulating hormones urging the “need to drink” [31]. Thirst perception was assessed using a Liker scale[32], [34] that ranged perceived thirst from 1 (“not thirsty at all”) to 9 (“very, very thirsty”).

### Statistical analysis

Descriptive values were provided for all the outcome variables. Engagement scores were non-normally distributed for all measures, as assessed by Shapiro-Wilk's test (p<0.05). A Spearman's rank-order correlation was run to assess the relationship between U_OSM_ and the rest of the hydration status markers (U_SG_, U_COL_, TPS and BIA). The size of the correlation was evaluated as follows; r<0.7 low; 0.7≤r<0.9 moderate; and ≥ 0.9 high [35]. Subjects were stratified according to their hydration status using U_OSMO_ values. A value of 700 mOsm·kg H_2_O^−1^ marks the limits between a correct hydration status and dehydration [1]. Thus, three intervals of equal amplitude (according to measurement units) were established according to the following cutoffs: from 250 to 700 mOsm·kg H_2_O^−1^ (euhydrated - G_1_), from 701 to 1.080 mOsm·kg H_2_O^−1^ (dehydrated - G_2_) and from 1.081 to 1.500 mOsm·kg H_2_O^−1^ (severely dehydrated - G_3_). Also, a Kruskal-Wallis test was performed between groups. Pairwise comparisons were performed using Dunn's [36] procedure with a Bonferroni correction for multiple comparisons.

## Results

**Figure 1:**
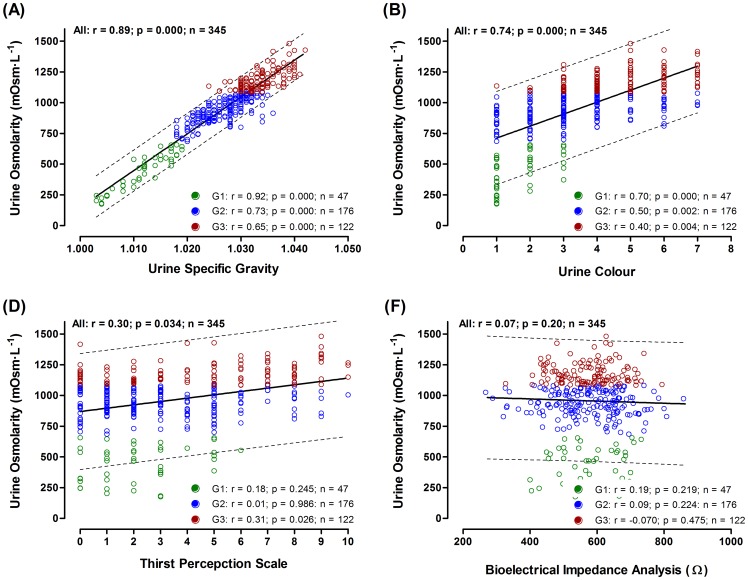
Correlation between U_OSM_ and U_SG_ (A), Urine color (B), Thirst perception scale (C) and Bioelectrical impedance analysis (D) in the whole sample and in each group. G1: U_OSM_ 250–700 mOsm·kg H_2_O^−1^; G2: U_OSM_ 701–1.080 mOsm·kg H_2_O^−1^; G3. U_OSM_ 1.081–1.500 mOsm·kg H_2_O^−1^.

Hydration status indexes were not different between males and females (U-Mann Whitney Wilcoxon test; p>0.05) or between sports (wrestling, taekwondo and boxing; Kruskal-Wallis test; p>0.05) and thus results are reported with all athletes as a group. A high linear and positive correlation was detected between U_SG_ and U_OSMO_ in the whole sample (r = 0.89; p = 0.000; n = 345). However, the correlation became lower as the dehydration status increased (G_1_ r = 0.92; p = 0.000; G_2_ r = 0.73; p = 0.000 and G_3_ r = 0.65; p = 0.000; Figure 1A).

The relationship between the U_OSMO_ and other hydration status markers was weak. U_COL_ showed a moderate although significant correlation when considering the whole sample (r = 0.743; p = 0.000) or the euhydrated group (G1: r = 0.702; p = 0.000). However, the correlation was low for the two dehydrated groups (G2: r = 0.498; p = 0.002; G3: r = 0.398; p = 0.004) (Figure 1B). TPS showed a significant but low correlation with the U_OSM_ in the whole sample and for G3 group (r<0.315 and r = 0.298, respectively; p<0.05) (Figure 1C). No significant correlation (p>0.05) was detected between the BIA assessments and U_OSM_ in any group (Figure 1D).

**Figure 2:**
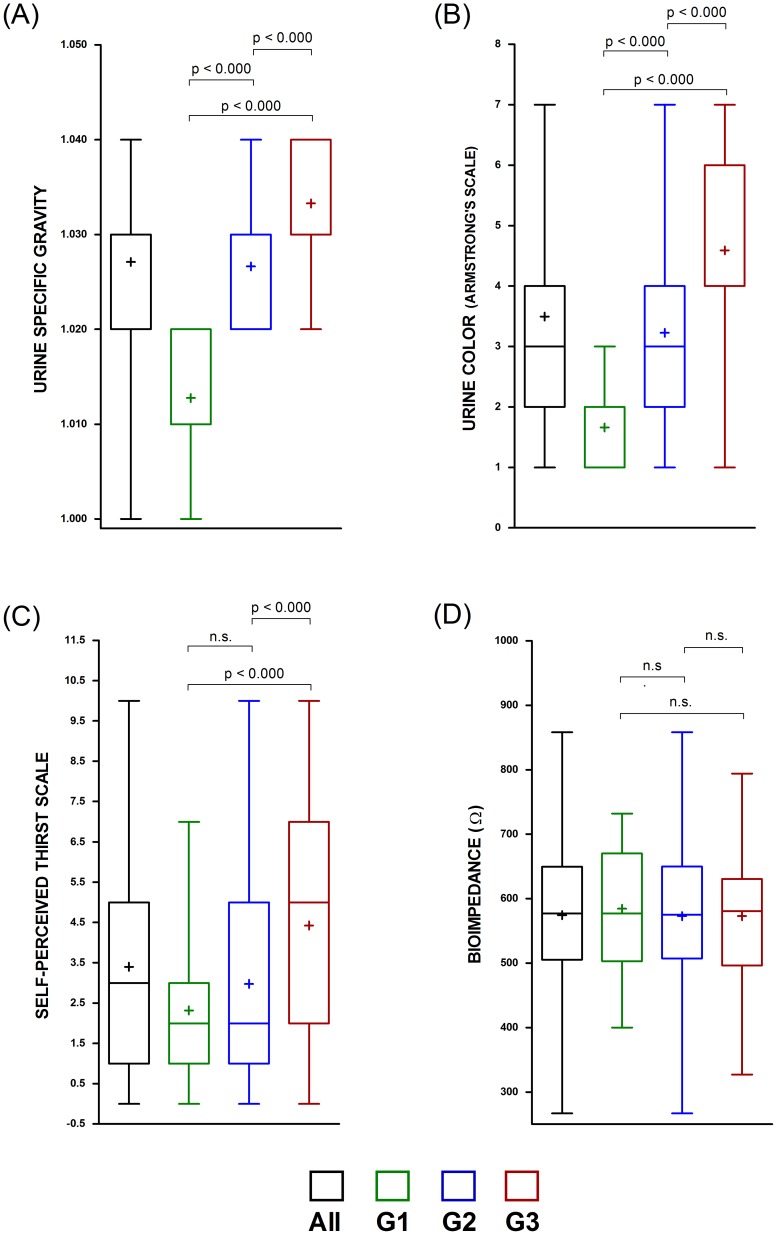
Descriptive values (whisker and box plot) and group differences according to U_OSMO_ status classification. Also, differences according to the Kruskal-Wallis test and Dunn's pairwise comparisons (Bonferroni correction for multiple comparisons).G1: U_OSM_ 250–700 mOsm·kg H_2_O^−1^; G2: U_OSM_ 701–1.080 mOsm⋅kg H_2_O^−1^; G3.U_OSM_ 1.081–1.500 mOsm·kg H_2_O^−1^.

Finally, a complementary Kruskal-Wallis analysis according to the athletes' dehydration status (euhydrated – G1, dehydrated – G2; and severely dehydrated –G3) reveals significant differences (p<0.05) between the 3 groups for the U_SG_ and U_COL_ methods. Nevertheless, the TPS cannot differ (p<0.05) between the first two groups (G1 and G2), and BIA do not distinguish (p<0.05) between any of the 3 groups (G1, G2 and G3) (Figure 2).

## Discussion

The current study compares different indexes of hydration status to urine osmolality (U_OSM_) as the gold standard non-invasive index [16], [23]. This comparison took place in a large sample of Olympic combat sports athletes (i.e. 345 athletes) during the official weigh-in of a real competition. While rapid reduction in body weight before competition is the easier non-invasive index of weight cutting thru dehydration it requires knowing what the “normal” weight of the athlete is. Referees and medical personnel at the competition arena do not have this information and thus require another index that is accurate, fast and non-invasive. Our aim was to determine which of the available non-invasive indexes (U_SG_; U_COL_; BIA and TPS) showed the better combination of sensibility to detect hypohydration, with affordability and simplicity in its use. This may prove useful to sport's governing bodies which are interested in preventing rapid weight loss during competition. Coaches and trainers can benefit too from easily assessing the degree of hypohydration in their combat athletes. We believe that fast and accurate identification of hypohydration is the first step into the prevention of weight cutting unhealthy practices.

While a similar question has been addressed in previous studies [14], [17], [18], [23], to our knowledge, this is the first study identifying the best non-invasive index using a large sample with a wide range of hydration statuses under a real competition situation. As a consequence of the real situation, we detected a large number of competitors with severe dehydration (176 samples with U_OSM_ above 701 mOsm·kg H_2_O^−1^ and 122 samples with U_OSM_ above 1.080 mOsm·kg H_2_O^−1^) beyond what has been previously reported [19], [37], [38]. Also, we observed that independent of sport discipline and gender a similar distribution of athletes were dehydrated or extremely dehydrated suggesting, as previously reported [37]–[41], that weight cutting is a broadly extended practice in Olympic combat sports.

Our results indicate that U_SG_ is the hydration index that better correlates with U_OSM_ (r = 0.89; p = 0.000; Figure 1) the assessment of U_SG_ being easier, cheaper and faster than that of U_OSM_. These results are consistent with the finding of Popowski et al [16] who compared the validity of U_SG_ and U_OSM_ to plasma osmolality, and concluded that both, U_SG_ and U_OSM_ correlate and are good measurements of hydration status. This data is also in agreement with results from our laboratory [21] reporting that U_SG_ is as sensitive as serum osmolality to detect 2 to 3% hypohydration. Based on the present results using an important sample size of elite athletes in a wide range of hydration statuses, we can substantiate that U_SG_ is a highly recommended index to assess hypohydration. Nevertheless, when dehydration increases U_SG_ presents lower correlation values (G2: r = 0.75; G3: r = 0.66; both p = 0.000; Figure 1). This validity decline, as body water loss increase, has been previously observed by Oppliger et al [24]. Nevertheless, dehydration is usually assessed based on a threshold value that is much below the values where U_SG_ starts to deviate from U_OSM_. Thus, a lowering in this correlation will rarely affect the classification of an individual as dehydrated or euhydrated.

Previous studies agreed that U_COL_ presents lower precision and accuracy values to determine the hydration status in humans compared to U_OSM_ and U_SG_ [17], [18], [25]. Nevertheless, different researchers consider that U_COL_ would be helpful in athletic, army or industrial settings where high precision assessment of body fluid deficit is not required [17], [22], [25]. Likewise, our data coincides in that U_COL_ is effective at discriminating different levels of dehydration (Figure 2) despite its lack of preciseness (G2: r = 0.498; p = 0.002, G3: r = 0.398; p = 0.004; Figure 1). As in previous studies, we can recommend U_COL_ analysis as an index to estimate hydration status of combat sports athletes; especially when water loss is not extreme. The low precision level of U_COL_ could be offset by its simplicity and low cost to assess hydration status on the field.

Some studies propose that BIA is a valid tool to assess hydration status in different populations [26], [27], [29]. However, our data suggests that BIA is not a good instrument to assess hydration level in combat sports athletes (Figure 1). In agreement with our results, other investigations argued that BIA may be a non-adequate instrument to evaluate exercise induced dehydration [28], [42]–[44]. Furthermore, our results show that during dehydration and severe dehydration (G2 and G3) BIA agreement with U_OSM_ worsens compared to euhydration (G1) (Figure 1). This is in accordance with the investigation of Asselin et al [45] which indicated that with dehydration levels of 2–3% of body mass, BIA standard equations failed to predict changes in total body water. As a limitation we used segmental BIA but mono-frequency analysis since, in our experience, this are the technical characteristics of the BIA equipment commonly found in combat sports clubs and high performance sports centers. Novel systems of BIA employ multi-frequency to determine the characteristics of the body fluids and tissues. Although they have shown even lower validity to estimate body composition [46], it has been recently reported that they are sensitive to evaluate acute dehydration in wrestlers [29]. It is unclear if the use of multi-frequency BIA could have increased its association with U_OSM_ in our data set.

Engell et al. [30] showed a high correlation between the perceived thirst and hypohydration before and after exercise in the heat. Young et al [33] agreed with this statement and added that using the 9 point scale (1 =  not at all thirsty; 9 =  very, very thirsty) a score between 3 and 5 could be a good indication that an individual is mildly dehydrated. Our results suggest that this perception scale is a valid indicator of hydration status, but only discriminating between euhydration and extreme dehydration. However, it does not distinguish low levels of dehydration from correct hydration (Figure 2). Maresh group [31], [32] provided data showing a thigh correlation between hypohydration and thirst perception when subjects are moderately dehydrated (i.e., ∼4%). It is known that numerous factors, apart from body water deficit, may alter the perception of thirst such us fluid palatability, time allowed for fluid consumption, time since last fluid ingestion, gastric distention, older age, gender, and heat acclimatization status[17]. Thus, while thirst perception may serve as an indicator of extreme dehydration, our data suggest that TPS is not accurate enough to correctly evaluate low and moderate levels of hypohydration during weight cutting in Olympic combat athletes.

Athletes involved in combat sports (e.g., wrestling, taekwondo and boxing) habitually weight-cut (i.e. weight loss through dehydration) to be included in a lower category at the official weigh-in before competition. Our study compares four different non-invasive hydration indexes (U_SG_, U_COL_, TPS, and BIA) to U_OSM_ as our gold standard non-invasive measure in a wide sample of competitive Olympic combat sports athletes. The aim is to find an alternative measure that, unlike U_OSM_, does not involve costly biochemical analysis and that can be readily used by sports medicine doctors, coaches and trainers on the combat arena. Our data suggests that U_SG_ is a good alternative to U_OSM_ since it highly correlates with U_OSM_, in conditions of low and severe dehydration (i.e. G2 and G3). However, U_COL_ can be an alternative and adequate tool to evaluate dehydration, especially if the dehydration level is not extreme. In contrast, our data discourages the use of TPS and BIA to measure hydration status after weigh-cut in combat sports athletes.
